# Multidimensional Clinical Surveillance of Pseudomonas aeruginosa Reveals Complex Relationships between Isolate Source, Morphology, and Antimicrobial Resistance

**DOI:** 10.1128/mSphere.00393-21

**Published:** 2021-07-14

**Authors:** Laura J. Dunphy, Glynis L. Kolling, Matthew L. Jenior, Joanne Carroll, April E. Attai, Farzad Farnoud, Amy J. Mathers, Molly A. Hughes, Jason A. Papin

**Affiliations:** a Department of Biomedical Engineering, University of Virginia, Charlottesville, Virginia, USA; b Division of Infectious Diseases and International Health, Department of Medicine, University of Virginia, Charlottesville, Virginia, USA; c Clinical Microbiology Laboratory, Department of Pathology, University of Virginia Health System, Charlottesville, Virginia, USA; d Department of Electrical and Computer Engineering, University of Virginia, Charlottesville, Virginia, USA; e Department of Biochemistry and Molecular Genetics, University of Virginia, Charlottesville, Virginia, USA; CDC

**Keywords:** infectious disease, antimicrobial resistance, clinical risk factors, *Pseudomonas aeruginosa*

## Abstract

Antimicrobial susceptibility in Pseudomonas aeruginosa is dependent on a complex combination of host and pathogen-specific factors. Through the profiling of 971 clinical P. aeruginosa isolates from 590 patients and collection of paired patient metadata, we show that antimicrobial resistance is associated with not only patient-centric factors (e.g., cystic fibrosis and antipseudomonal prescription history) but also microbe-specific phenotypes (e.g., mucoid colony morphology). Additionally, isolates from different sources (e.g., respiratory tract, urinary tract) displayed rates of antimicrobial resistance that were correlated with source-specific antimicrobial prescription strategies. Furthermore, isolates from the same patient often displayed a high degree of heterogeneity, highlighting a key challenge facing personalized treatment of infectious diseases. Our findings support novel relationships between isolate and patient-level data sets, providing a potential guide for future antimicrobial treatment strategies.

**IMPORTANCE**P. aeruginosa is a leading cause of nosocomial infection and infection in patients with cystic fibrosis. While P. aeruginosa infection and treatment can be complicated by a variety of antimicrobial resistance and virulence mechanisms, pathogen virulence is rarely recorded in a clinical setting. In this study, we discovered novel relationships between antimicrobial resistance, virulence-linked morphologies, and isolate source in a large and variable collection of clinical P. aeruginosa isolates. Our work motivates the clinical surveillance of virulence-linked P. aeruginosa morphologies as well as the tracking of source-specific antimicrobial prescription and resistance patterns.

## INTRODUCTION

Pseudomonas aeruginosa is a highly versatile human pathogen (1) and a leading cause of nosocomial infection (2, 3) and infection in patients with cystic fibrosis (CF) (4, 5). P. aeruginosa has the ability to rapidly develop multidrug resistance (6, 7) and can infect diverse host niches, including the respiratory tract (8), the urinary tract (9, 10), wounds (11), and the bloodstream (12, 13). In addition to challenges surrounding antimicrobial resistance, clearance of P. aeruginosa infection can be hindered by the production of virulence factors (e.g., alginate and pyocyanin), which can impede antimicrobial treatment (14), dampen the host immune response (15), and otherwise promote pathogen survival (8). Because of the unique intrinsic antimicrobial resistance to many antimicrobials and the ability for resistance to emerge, P. aeruginosa infections can be further complicated in patients with a history of antimicrobial treatment (16–18) or in those with polymicrobial infections (19, 20).

Despite the breadth of P. aeruginosa infections observed clinically, surveillance studies are typically limited to specific patient cohorts (e.g., CF patients and ICU patients) or a small number of isolate sources (e.g., lung and burn wound) (21–24). Additionally, although others have profiled morphological phenotypes in clinical isolates (22, 25, 26), this is not common practice, and pathogen-specific characteristics beyond susceptibility profiles are rarely available in electronic health records (EHRs). These limitations in surveillance practices and EHRs have made it difficult to gain a holistic perspective of P. aeruginosa infections across isolate sources, comorbidities, and other host factors that can influence microbial phenotypes.

Here, we indiscriminately collected clinical P. aeruginosa isolates from the University of Virginia (UVA) hospital from February 2019 to February 2020. Patient metadata, antimicrobial prescription history, and bacterial susceptibility profiles were recorded for 971 isolates from 590 patients. In addition to these clinical measures, we profiled four virulence-linked morphological phenotypes that could be rapidly characterized with minimal passaging: mucoid phenotype, metallic sheen, pigment production, and hemolytic activity. These phenotypes have been previously supported to indicate respective production of alginate (14), quorum-sensing molecules (27), siderophores (28), toxins (15), and the hemolytic enzyme phospholipase C (29). With these data, we were able to identify clinical and microbiological factors associated with antimicrobial susceptibility or resistance. Separating the data by isolate source revealed source-specific phenotypic and susceptibility patterns as well as source-specific prescription histories. By providing an integrated view of relationships between patient demographics, antimicrobial prescription history, isolate source, isolate morphology, and antimicrobial susceptibility or resistance in P. aeruginosa infection, we aim to improve data-driven treatment of bacterial infections.

## RESULTS

### Multidimensional profiling of Pseudomonas aeruginosa clinical isolates.

**FIG 1:**
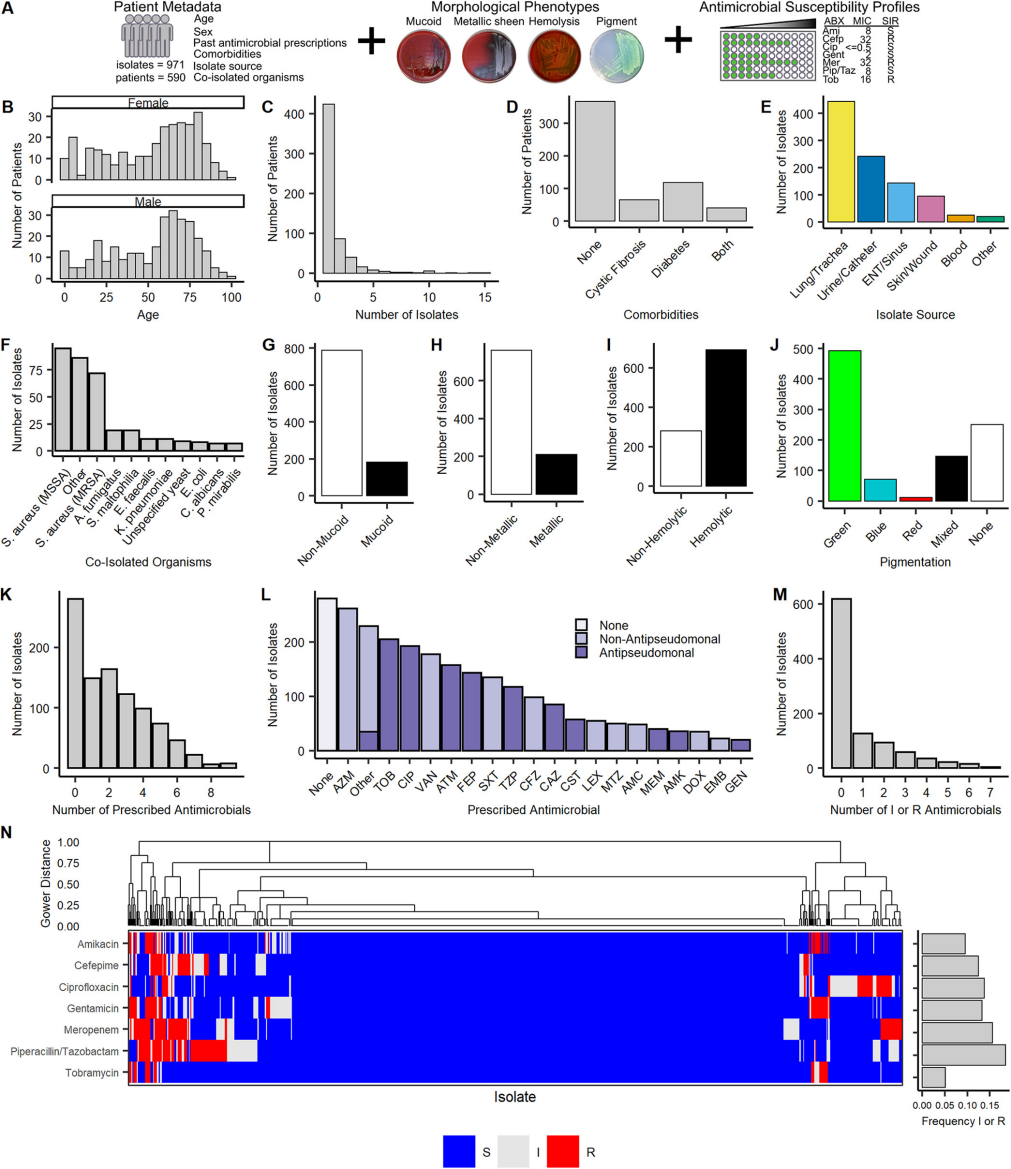
A total of 971 clinical Pseudomonas aeruginosa isolates from 590 patients collected during 1 year at the UVA Health System. (A) Outline of data types collected. (B) Age distributions of 590 patients separated by sex. (C) Histogram of the number of isolates collected per patient. (D) Number of patients with cystic fibrosis or diabetes. (E) Distribution of isolate sources. (F) Distribution of coisolating pathogens identified at the time of P. aeruginosa isolation. Pathogens not in the top 10 most common are accounted for in “Other.” All isolates not shown had no coisolating pathogens on the day of P. aeruginosa isolation. (G) Distribution of mucoid and nonmucoid isolates on blood agar. (H) Distribution of isolates with and without a metallic sheen on blood agar. (I) Distribution of hemolytic and nonhemolytic isolates on blood agar. (J) Distribution of pigment production on cetrimide agar. (K) Number of unique antimicrobials the patient of each isolate was prescribed prior to isolate collection. Multiple prescriptions of the same antimicrobial are counted as a single prescription. (L) Number of isolates from patients prescribed each antimicrobial prior to isolate collection. Multiple prescriptions of the same antimicrobial are counted as a single prescription. Antimicrobial abbreviations can be found in Table S3 and are consistent with those outlined by the American Society for Microbiology with the exception of dapsone (DDS), imipenem/cilastatin (IPMC), metronidazole (MTZ), and rifaximin (RIFX), which were excluded from these guidelines. Antimicrobials prescribed fewer than 20 times were grouped into “Other.” Colors reflect antipseudomonal activity. (M) Distribution of the number of resistant or intermediate antimicrobial susceptibility results per isolate. Susceptibility testing was performed on seven antimicrobials for each isolate. (N) Susceptibility profiles of all isolates and population-level frequencies of resistance or intermediate resistance to each measured antimicrobial. Susceptibility profiles were clustered by Gower distance with complete linkage. Colors reflect susceptibility to each measured antimicrobial (S, susceptible; I, intermediate; R, resistant).

Between 10 February 2019 and 21 February 2020, we obtained clinical isolates of P. aeruginosa from the UVA Health System Clinical Microbiology Laboratory, which collects samples from a range of outpatient and inpatient settings in the region. This was approximately 60% of all P. aeruginosa isolates identified by the UVA Clinical Microbiology Laboratory during this time period. For each isolate, we recorded associated patient metadata, bacterial morphological phenotypes, and antimicrobial susceptibility profiles (Fig. 1A). In total, we collected complete profiles for 971 isolates from 590 patients within the UVA Health System.

10.1128/mSphere.00393-21.7 TABLE S3 Complete list of antimicrobials considered in this study arranged by antimicrobial class in descending frequency of prescription. Download Table S3, DOCX file, 0.02 MB. Copyright © 2021 Dunphy et al. 2021 Dunphy et al. https://creativecommons.org/licenses/by/4.0/ This content is distributed under the terms of the Creative Commons Attribution 4.0 International license.

Patient age, sex, comorbidities (CF and diabetes mellitus), and isolate source were collected from the EHR to better understand the patient demographics of P. aeruginosa infection. To account for fundamental differences in the CF population, CF isolates were separated from non-CF isolates where necessary in downstream analyses. Patients ranged from 1.5 months old to 98 years old, and the total population had a median (interquartile range [IQR]) age of 60 (31.1 to 73) (Fig. 1B; Table S1). Multiple isolates from the same patient were retained if the isolates were collected on different days or if they could be distinguished by morphology or antimicrobial susceptibility. The majority of patients had a single P. aeruginosa isolate identified during the year, while 166 patients had two or more isolates and three patients had 10 or more isolates sampled during the course of the year (Fig. 1C). Isolates from CF patients were overrepresented, accounting for 34.4% (334/971) of all isolates while CF patients made up only 18.0% (106/590) of the patient population surveyed (Fig. 1D; Table S1). Isolates from the lung/trachea (e.g., sputum, bronchoscopy and tracheal aspirate) were most common, accounting for 45.6% (443/971) of isolates. Respiratory isolates were followed in occurrence by isolates obtained from the urine/catheter (24.9%), ear/nose/throat (ENT)/sinus (14.8%), skin/wound (9.9%), blood (2.7%), and all other sources (2.1%) (Fig. 1E; Table S2).

10.1128/mSphere.00393-21.5 TABLE S1 Patient and isolate metadata. Download Table S1, DOCX file, 0.01 MB. Copyright © 2021 Dunphy et al. 2021 Dunphy et al. https://creativecommons.org/licenses/by/4.0/ This content is distributed under the terms of the Creative Commons Attribution 4.0 International license.

10.1128/mSphere.00393-21.6 TABLE S2 Breakdown of isolate sources and antimicrobial susceptibility of 971 clinical isolates. Download Table S2, DOCX file, 0.01 MB. Copyright © 2021 Dunphy et al. 2021 Dunphy et al. https://creativecommons.org/licenses/by/4.0/ This content is distributed under the terms of the Creative Commons Attribution 4.0 International license.

To understand clinical and microbiologic coassociations with other bacteria, we searched patient EHRs for organisms coisolated on the same day as P. aeruginosa. The majority of cultures (68.8%; 668/971) were monomicrobial (i.e., no other pathogen was isolated from the same source on that day). The most common copathogens were methicillin-sensitive Staphylococcus aureus (MSSA) and methicillin-resistant S. aureus (MRSA) (Fig. 1F). MSSA and MRSA were predominantly isolated from the lung/trachea and ENT/sinus (Fig. S1A).

10.1128/mSphere.00393-21.1 FIG S1 Coisolating organisms by isolate source and most common isolate susceptibility and morphological profiles. (A) All coisolating organisms grouped by isolate source. (B to D) The 10 most common complete profiles (B), resistance profiles (C), and morphological profiles (D) seen across all 971 isolates. Histograms provide number of isolates with each profile. Resistance profiles exclude isolates that were susceptible to all antimicrobials. Susceptibility profiles include SIR calls (blue, susceptible; white, intermediate; red, resistant) for seven antimicrobials. Morphological profiles include presence (black) or absence (white) of the mucoid phenotype, metallic sheen, and hemolysis on blood agar, and observed pigment production on cetrimide agar (green, green pigment; blue, blue pigment; brown, mixed pigment; white, no pigment). Download FIG S1, TIF file, 2.4 MB. Copyright © 2021 Dunphy et al. 2021 Dunphy et al. https://creativecommons.org/licenses/by/4.0/ This content is distributed under the terms of the Creative Commons Attribution 4.0 International license.

Four morphological phenotypes were measured for each isolate: mucoid phenotype, metallic sheen, hemolysis, and pigment production. The majority of isolates were nonmucoid (788/971; 81.2%), nonmetallic (760/971; 78.3%), and hemolytic (690/971; 71.1%) on blood agar and produced green pigment on cetrimide agar (493/971; 50.8%) (Fig. 1G to J). This combination of morphologies was also the most common phenotypic profile across isolates in this study (Fig. S1B and D).

For each isolate, we recorded all antimicrobials prescribed to the given patient up to 60 days prior to sample isolation. This window was chosen to assess the relationship between recent antimicrobial prescription and resistance. We recorded prescriptions of antipseudomonal (AP) antimicrobials and nonantipseudomonal (NAP) antimicrobials (Table S3). If a patient was prescribed the same antimicrobial multiple times during the 60-day period, this was counted as a single antimicrobial prescribed. The top five antimicrobials prescribed across all isolates in this study included azithromycin (NAP), tobramycin, ciprofloxacin, vancomycin (NAP), and aztreonam (Fig. 1L). Overall, the majority of isolates (691/971; 71.2%) were identified in patients who were recently prescribed antimicrobials, while only 280 (28.8%) isolates were from patients with no antimicrobial use in the last 60 days (Fig. 1K).

Finally, we assessed antimicrobial susceptibility across all 971 clinical P. aeruginosa isolates. Based on measured MICs, isolates were classified in susceptibility profiles as susceptible (S), intermediate (I), or resistant (R) to seven antimicrobials: amikacin, cefepime, ciprofloxacin, gentamicin, meropenem, piperacillin-tazobactam, and tobramycin. As isolates are not guaranteed to be entirely susceptible, intermediate resistance was classified as resistance unless otherwise stated. Piperacillin-tazobactam had the highest frequency of resistance (18.4%), while tobramycin had the lowest frequency of resistance (5.0%) (Fig. 1N; Table S2). The majority of isolates (617/971; 63.5%) were susceptible to all seven antimicrobials tested across all isolates (Fig. 1M and N; Fig. S1B and C). Aztreonam susceptibility was additionally measured for 751 isolates, of which 85.2% (640/751) were susceptible. In total, 13.8% of isolates (134/971) were multidrug resistant (MDR), where isolates were considered MDR if they were intermediate or resistant to one or more antimicrobial agents in at least three antimicrobial categories (30).

### Logistic regression identifies variables associated with antimicrobial resistance.

**FIG 2:**
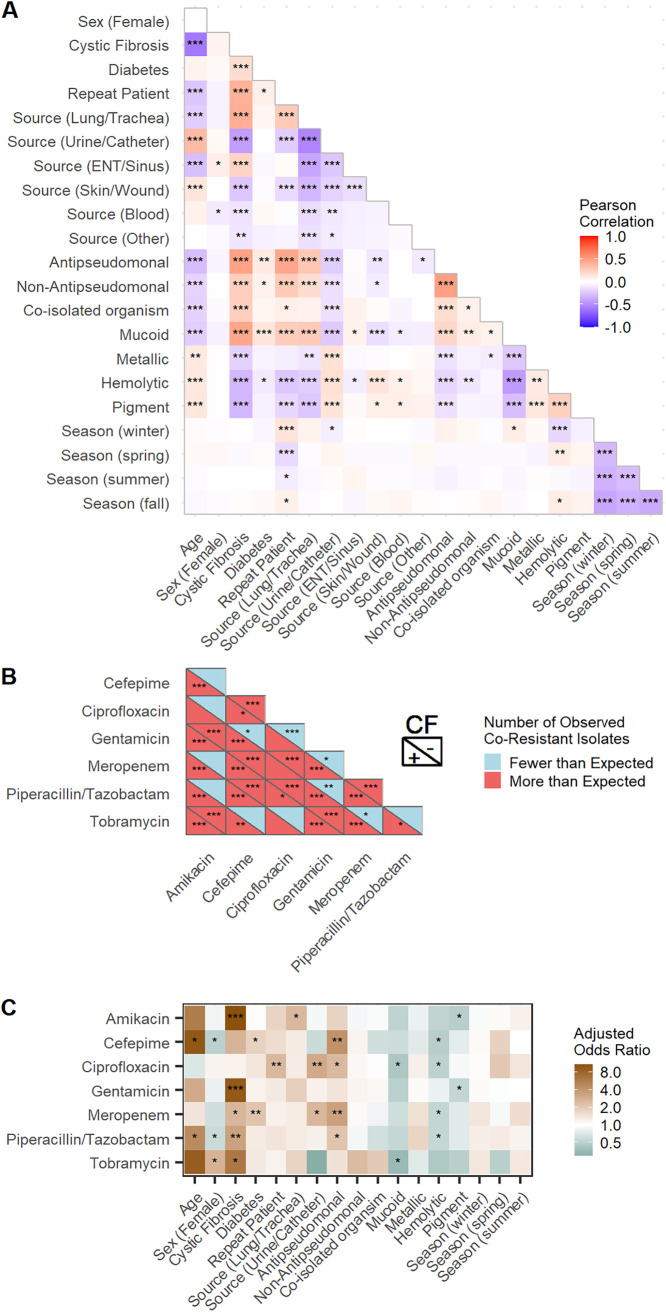
Pairwise relationships between demographic, phenotypic, and antimicrobial susceptibility data. (A) Pearson correlation coefficients between different phenotypic and demographic measurements of all clinical isolates. Asterisks denote BH-corrected *P* values (*, *P* < 0.05; **, *P* < 0.01; ***, *P* < 0.001). (B) Observed number of coresistant isolates relative to the number that would be expected based on the resistance frequencies of individual antimicrobials, determined for isolates from patients with (bottom) and without (top) CF. Intermediately resistant isolates were considered resistant. Asterisks denote BH-corrected *P* values from Fisher's exact test for statistical independence (*, *P* < 0.05; **, *P* < 0.01; ***, *P* < 0.001). (C) Adjusted odds ratios between measured features and susceptibility data across all isolates (a ratio of >1 denotes increased odds of resistance; a ratio of <1 denotes decreased odds of resistance). Adjusted odds ratios were calculated from logistic regression models (see Materials and Methods for model descriptions and Data Set S1 for regression coefficients). Asterisks denote BH-corrected *P* values (*, *P* < 0.05; **, *P* < 0.01; ***, *P* < 0.001).

To better understand trends across our data, we examined pairwise relationships between and across patient variables, morphological measures, and susceptibility profiles. First, we identified positively and negatively correlated patient and isolate-specific features (Fig. 2A; Data Set S1). As expected, CF was negatively correlated with age, with a median (IQR) age of 25 (19 to 36.8), and positively correlated with repeat infection, diabetes, recent antimicrobial prescription (AP and NAP), mucoid phenotype, and lung/trachea and ENT/sinus sources (*P* < 0.001). Notably, correlation patterns varied by isolate source. For example, skin/wound and urine/catheter isolates positively correlated with hemolytic activity and negatively correlated with the mucoid phenotype, while lung/trachea isolates displayed correlations in the opposite directions (*P* < 0.001). Seasonality, patient sex, and diabetes were not strongly correlated with other measured variables. While some pairwise correlations may have been confounded by strong covariates such as CF, the consistent co-occurrence of specific isolate sources and corresponding morphological characteristics strongly indicates that these phenotypes contribute in some way to success of the pathogen under each condition.

10.1128/mSphere.00393-21.9 DATA SET S1 CSV of adjusted *P* values, fractional deviations, correlation coefficients, and adjusted odds ratios. Download Data Set S1, CSV file, 0.05 MB. Copyright © 2021 Dunphy et al. 2021 Dunphy et al. https://creativecommons.org/licenses/by/4.0/ This content is distributed under the terms of the Creative Commons Attribution 4.0 International license.

We next quantified the number of isolates that were coresistant to every pairwise combination of antimicrobials in both CF and non-CF subpopulations. The expected number of coresistant isolates was calculated for each antimicrobial pair using population-level resistance frequencies of each drug, assuming statistical independence. Across both CF and non-CF isolates, coresistance was observed at a higher frequency than would be expected across all pairs of antimicrobials of the same class (aminoglycosides: amikacin, gentamicin, and tobramycin; beta-lactams: cefepime, meropenem, and piperacillin-tazobactam) (*P* < 0.001) (Fig. 2B). While we and others (31) observed instances of coresistance across pairs of beta-lactam and aminoglycoside antimicrobials in CF isolates (*P* < 0.01), this trend was not observed in non-CF isolates. Differences in coresistance frequencies between CF and non-CF isolate populations may be impacted by source-specific nutrient environments and various patterns of antimicrobial exposures. For example, isolates in the CF lung environment are more likely to have been exposed to multiple antimicrobials for prolonged periods of time.

Finally, we identified patient and isolate-specific features that were associated with antimicrobial resistance. Multiple logistic regression is a supervised machine learning approach that allows for isolation and assessment of associations between multiple inputs and a binary output and reports an odds ratio for the strength of each relationship. Logistic regression models were built to measure the association of demographic and morphological variables with resistance to each tested antimicrobial (Fig. 2C; Data Set S1; also, see Materials and Methods). Adjusted odds ratios were calculated from the coefficients of each logistic regression model. The adjusted odds ratio describes the odds that a given input is associated with antimicrobial resistance, independent of all other inputs. This minimizes the confounding effects of other inputs in the model (e.g., measures association of age independent of CF status). While odds ratios are not guaranteed to be causal, they can be used to help identify risk factors for antimicrobial resistance (10, 32).

Consistent with previous literature, increasing age, CF, diabetes, and prior antipseudomonal antimicrobial prescription were each associated with resistance to multiple antimicrobials, indicated by significant adjusted odds ratios greater than 1 (*P* < 0.05) (Fig. 2C; Data Set S1) (16, 33–35). We observed multiple associations between morphological phenotypes and antimicrobial resistance that were not expected. First, beta-lactam resistance was associated with a lack of hemolytic ability (*P* < 0.05). Second, both amikacin resistance and gentamicin resistance were associated with a lack of pigment production (*P* < 0.05). Third, tobramycin resistance was less likely to be seen in mucoid isolates (*P* < 0.05). Demographically, females were associated with decreased odds of cefepime and piperacillin-tazobactam resistance and increased odds of tobramycin resistance (*P* < 0.05). The presence of coisolated organisms, recent nonantipseudomonal antimicrobial prescriptions, metallic sheen, and seasonality were not significantly associated with antimicrobial resistance (*P* > 0.05). Overall, we confirm previously observed risk factors of antimicrobial resistance and identify novel potential associations between resistance and morphological phenotypes that are independent of CF status and other demographic variables included in our models.

### P. aeruginosa morphology and susceptibility patterns are associated with isolate source.

**FIG 3:**
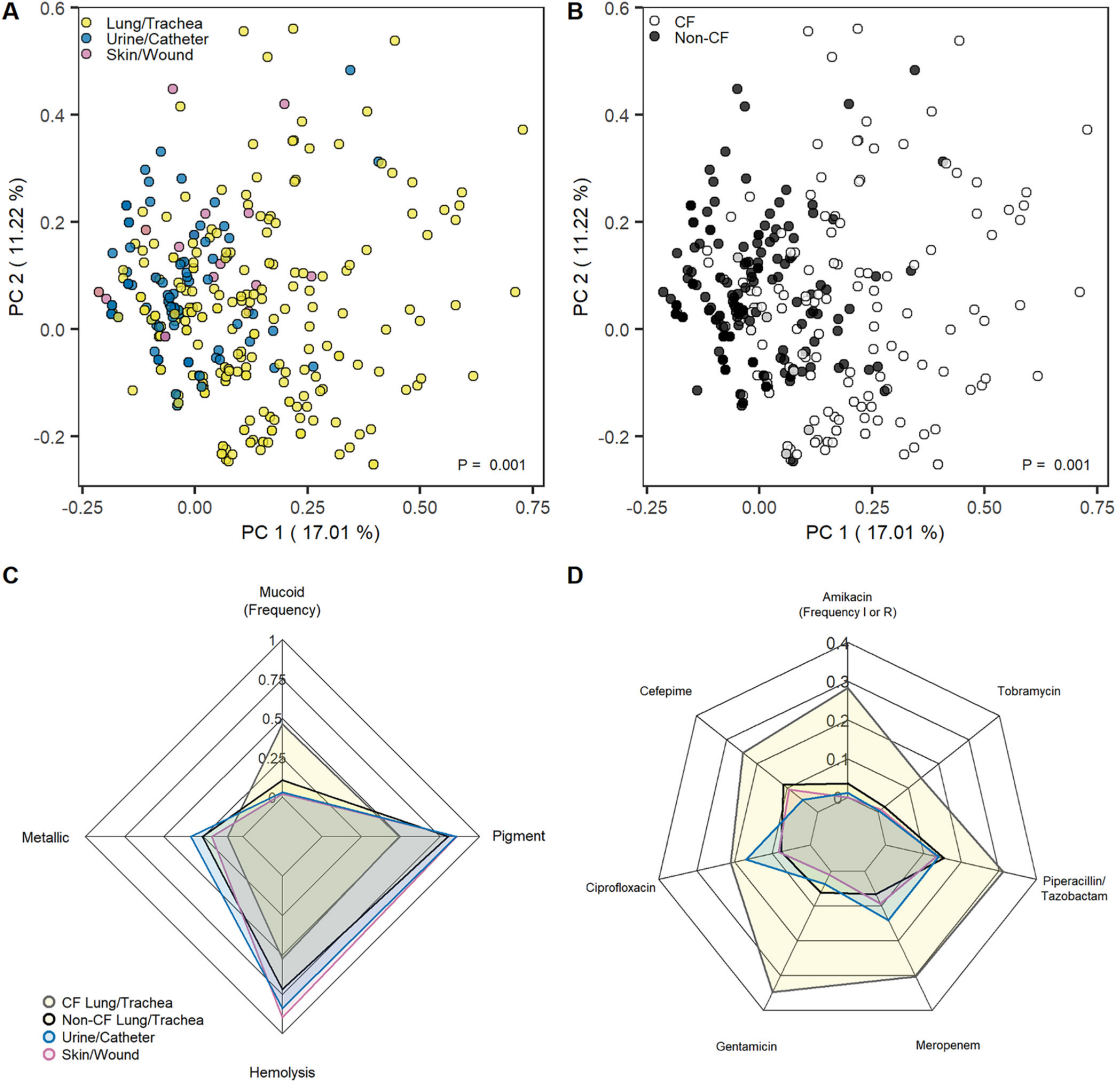
P. aeruginosa morphologies and antimicrobial susceptibility profiles vary across isolate sources. PCoA of Gower distances between isolate susceptibility profiles and phenotype profiles colored by isolate source (A) and whether a patient had CF (B). Significant differences were calculated by PERMANOVA. Radar plots show the fraction of phenotype-expressing (C) or intermediate and resistant (D) isolates. CF lung isolates (gray, yellow fill), non-CF lung isolates (black, yellow fill), skin/wound isolates (pink), and urine/catheter isolates (blue) are compared.

While P. aeruginosa research is dominated by studies of respiratory infections in CF patients and burn wound models, P. aeruginosa can infect many regions of the body (16). We indiscriminately gathered P. aeruginosa isolates from all sources and patients that came through the UVA Health System, allowing comparisons between commonly studied sources (e.g., CF lung, skin/wound), and understudied but commonly occurring sources such as in urine and catheters. Principal-coordinate analysis (PCoA) is an unbiased clustering approach that can be performed on any distance matrix (36). PCoA a preferred alternative to principal-component analysis (PCA) for clustering of categorical data because it does not require the use of Euclidean distances. Clustering of categorical morphological phenotype and susceptibility data via PCoA revealed that lung/trachea isolates were more distant than urine/catheter and skin/wound isolates (*P* < 0.001), which generally clustered together (Fig. 3A; Fig. S2A). The same PCoA projection labeled by CF lung/trachea isolates and non-CF lung/trachea, urine/catheter, and skin/wound isolates revealed that the majority of the total lung/trachea dispersion was driven by CF patients (Fig. 3B; Fig. S2B). These results indicate that CF lung/trachea isolates display greater multivariate heterogeneity than isolates from other sources, highlighting the diversity of adaptations that P. aeruginosa may be able to undergo in the lungs of these patients.

10.1128/mSphere.00393-21.2 FIG S2 Distributions of isolate-isolate distances within PCoA clusters. Distributions of Gower distances between isolates from the same source (A) or with the same cystic fibrosis status (B). The crossbar denotes median Gower distances between isolates from the same source. Pairwise comparisons were made with by Wilcoxon rank sum test. Asterisks denote BH-corrected (A) or uncorrected (B) *P* values (***, *P* < 0.001). Download FIG S2, TIF file, 1.4 MB. Copyright © 2021 Dunphy et al. 2021 Dunphy et al. https://creativecommons.org/licenses/by/4.0/ This content is distributed under the terms of the Creative Commons Attribution 4.0 International license.

Differences between CF lung/trachea isolates and isolates from other sources were also seen in population-level frequencies of isolate morphologies and resistances. Non-CF lung/trachea isolates, urine/catheter isolates, and skin/wound isolates displayed relatively similar frequencies of all four measured morphological features. Conversely, CF lung/trachea isolates had an increased rate of the mucoid phenotype (37, 38) and decreased rates of all other phenotypes relative to the other three isolate sources (Fig. 3C). In addition to phenotypic differences, CF lung/trachea isolates had increased rates of resistance to all tested antimicrobials (Fig. 3D). All other isolate sources had comparable rates of antimicrobial resistance, with the exception of urine/catheter isolates, which had elevated rates of ciprofloxacin and meropenem resistance. This result was consistent with associations seen in logistic regression (Fig. 2C). Overall, we observed considerable divergence between CF lung/trachea samples and all other isolates as well as elevated frequencies of resistance to multiple antimicrobials in urine/catheter isolates relative to other non-CF sources.

### Antimicrobial prescription history is associated with isolate source.

**FIG 4:**
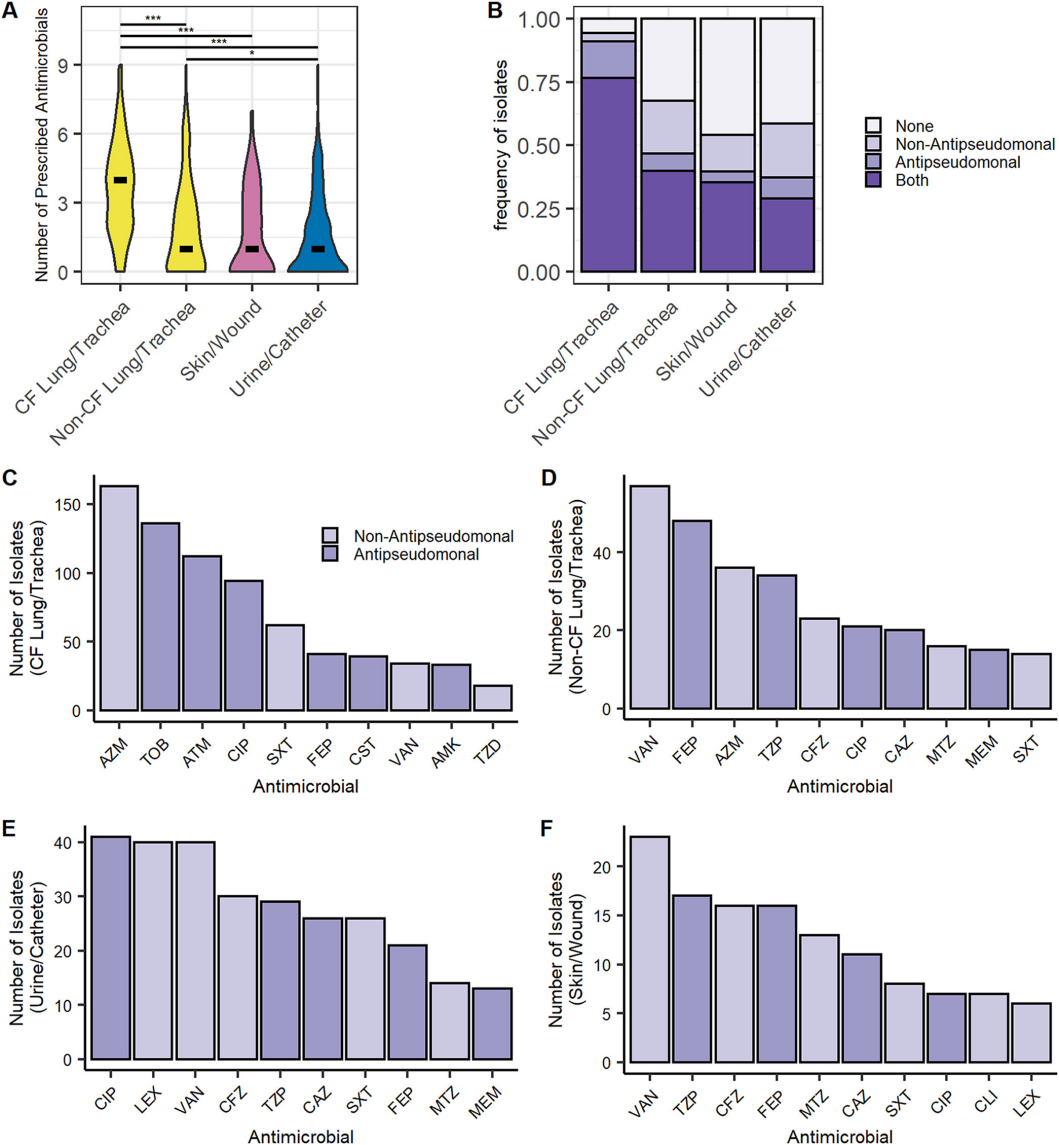
Isolate antimicrobial prescription history varies by source. (A) Number of antimicrobials prescribed prior to isolate collection by source. Multiple prescriptions of the same antimicrobial are considered a single prescription. The crossbar indicates the median number of prescriptions for that isolate source. Pairwise comparisons were made by Wilcoxon rank sum test. Asterisks denote BH-corrected *P* values (*, *P* < 0.05; ***, *P* < 0.001). (B) Frequency of isolates prescribed no antimicrobials, antipseudomonal antimicrobials, nonantipseudomonal antimicrobials, or both antipseudomonal and nonantipseudomonal antimicrobials prior to isolate collection by source. (C to F) Top 10 most common antimicrobials prescribed prior to isolate collection from CF lung/trachea (C), non-CF lung/trachea (D), urine/catheter (E), and skin/wound (F).

Our study and others have shown that previous antipseudomonal antimicrobial prescription is associated with antimicrobial resistance (Fig. 2C) (17, 35). To better understand source-specific differences in P. aeruginosa isolates, we compared patient antimicrobial prescription history across isolate sources. CF lung/trachea isolates came from patients prescribed significantly (*P < *0.001) more antimicrobial treatments than other sources (Fig. 4A). While prior prescription of nonantipseudomonal antimicrobials was not a risk factor for antimicrobial resistance, antipseudomonal antimicrobial prescription was a risk factor for cefepime, meropenem, piperacillin-tazobactam, and ciprofloxacin resistance (Fig. 2C). Nearly all CF lung/trachea isolates (91.1%) came from patients who had recently been prescribed at least one antipseudomonal. Antipseudomonal antimicrobial prescription in relation to CF lung/trachea isolates was increased relative to other isolate sources, which had frequencies between 37.2% and 46.7% (Fig. 4B). Urine/catheter isolates had the highest frequency of only nonantipseudomonal coverage (21.5%). CF lung/trachea isolates were more likely to have come from patients on multiple antimicrobials and to have had recent prescriptions for one or more antipseudomonal antimicrobials. Combined, these trends offer a rational explanation for the high frequency of antimicrobial resistance observed in CF lung/trachea isolates relative to isolates from other sources.

To better understand differences in prescription history across isolate sources, we identified the top 10 most common antimicrobials prescribed prior to P. aeruginosa isolation from each source (Fig. 4C to F). Antimicrobials common in CF lung/trachea isolates, including tobramycin, aztreonam, colistin, and amikacin, were not among the most prescribed antimicrobials of other sources (Fig. 4C). Conversely, there was a high degree of overlap in commonly prescribed antimicrobials across the other three sources (Fig. 4D to F). Notably, ciprofloxacin was the most common antipseudomonal treatment prescribed prior to isolation of P. aeruginosa from the urine/catheter (Fig. 4E). The frequent use of ciprofloxacin in urine/catheter and CF lung/trachea isolates could explain increased rates of ciprofloxacin resistance in these isolate sources. Combined, our results indicate that CF lung/trachea isolates varied in morphology, susceptibility profile, and antimicrobial prescription history relative to other common sources of isolates. Conversely, with the exception of ciprofloxacin resistance in urine/catheter isolates, morphological phenotypes and susceptibility profiles across other compared isolate sources remained relatively conserved.

### Intrapatient isolates display various levels of heterogeneity.

**FIG 5:**
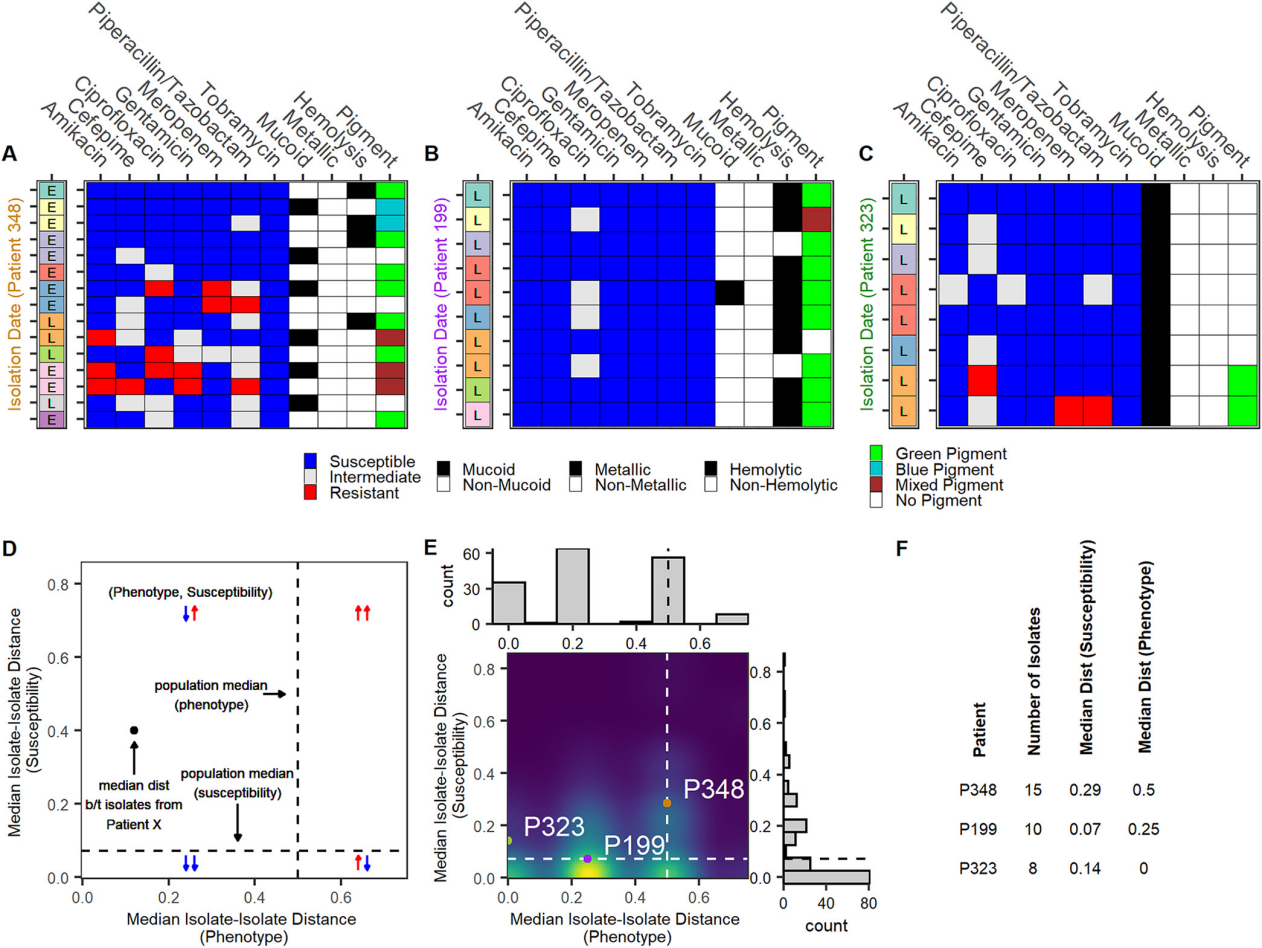
Intrapatient isolates display heterogeneous phenotypes and antimicrobial susceptibility profiles. (A to C) Susceptibility and morphological profiles of isolates from patient 348 (A), patient 199 (B), and patient 323 (C) collected over a 1-year period. Patient ages were within 3 years of one another. Isolates are arranged in chronological order of collection with the earliest isolate on top (left labels: L, lung/trachea; E, ENT/sinus). Susceptibility to seven antimicrobials (blue, susceptible; white, intermediate; red, resistant), presence (black) or absence (white) of the mucoid phenotype, metallic sheen, and hemolysis on blood agar, and observed pigment production on cetrimide agar (green, green pigment; blue, blue pigment; brown, mixed pigment; white, no pigment). (D) Key to density of median intrapatient isolate-isolate distances. Dashed lines denote median isolate-isolate distances across all isolate pairs. Aside from population medians, median distances were measured across isolate pairs within isolates from the same patient. Arrows indicate whether patients in a given quadrant have higher (red) or lower (blue) variability in phenotype (first arrow) or susceptibility (second arrow) relative to the whole population. (E) Density of median intrapatient isolate-isolate distances. Gower distances were calculated separately for morphological profiles and susceptibility profiles. Areas of high patient density are shown in yellow. Dashed lines denote the median distances across all isolate pairs. Data for patient 348 (orange), patient 199 (purple), and patient 323 (green) are highlighted. (F) Number of isolates per patient and median isolate-isolate distances.

Having assessed population- and source-level patterns, we lastly examined intrapatient variability in phenotypes and susceptibility profiles. While the majority of patients had one isolate associated with them during the collection period, 166 patients contributed multiple isolates over one or more visits (Fig. 1C). Of these 166 patients, 74 of them had cystic fibrosis. We observed that even patients close in age and with similarly complex antimicrobial prescription histories could have widely differing isolate profiles (Fig. 5A to C and F; Fig. S3). Particularly, individual patients could have multiple isolates expressing a high degree of variability in phenotype and/or susceptibility profiles (Fig. 5A and C).

10.1128/mSphere.00393-21.3 FIG S3 Antimicrobial prescription histories for three patients. History of antimicrobial prescriptions in the 60 days prior to isolate collection for isolates from patient 348 (A), patient 199 (B), and patient 323 (C). Isolates are arranged in chronological order of collection with the earliest isolate on top (left). Prescriptions are colored by antipseudomonal activity. Isolates collected on the same date have identical prescription histories. Download FIG S3, TIF file, 1.4 MB. Copyright © 2021 Dunphy et al. 2021 Dunphy et al. https://creativecommons.org/licenses/by/4.0/ This content is distributed under the terms of the Creative Commons Attribution 4.0 International license.

To more globally measure intrapatient heterogeneity, for each patient, two median isolate-isolate distances were calculated: one across morphological phenotype data and one across antimicrobial susceptibility data. These values were compared to distances across all isolate pairs from all patients (Fig. S4). Patients with a distance less than the median of the population were considered to have less variability than the entire population at large (Fig. 5D). Contrary to what might be expected, only 33.1% (55/166) of patients had less variation in both isolate phenotypes and susceptibility data than the population at large (Fig. 5D and E, bottom left quadrants). The remaining 66.9% (111/166) of patients had isolates at least as heterogeneous as those across the population at large in either one or both distance measures (Fig. 5D and E: top left, top right, and bottom right quadrants, inclusive of quadrant boundaries). In total, 37 patients (22.3%) had isolate profiles that were at least as heterogeneous as across the population at large (Fig. 5D and E, top right quadrants). Across all isolates, phenotypic distance weakly correlated with susceptibility distance (Pearson correlation coefficient = 0.12; *P* < 0.001). Consistent with literature, these findings suggest that at a patient level, P. aeruginosa isolates can be highly heterogeneous (26, 39). Additional studies are required to determine whether phenotypic heterogeneity correlates with heterogeneity in susceptibility profiles.

10.1128/mSphere.00393-21.4 FIG S4 Isolate-isolate distance matrix. Gower distances between all isolate pairs. Distance calculated on isolate susceptibility and morphological profiles. Distance matrix clustered by hierarchical clustering with complete linkage. Gower distance of 0 indicates that two isolates had identical profiles. Source of each isolate is shown. Download FIG S4, TIF file, 1.4 MB. Copyright © 2021 Dunphy et al. 2021 Dunphy et al. https://creativecommons.org/licenses/by/4.0/ This content is distributed under the terms of the Creative Commons Attribution 4.0 International license.

## DISCUSSION

This study presents a multidimensional snapshot of a year of clinical P. aeruginosa isolates from a single health care system from February 2019 to February 2020. Although P. aeruginosa is known for its ability to develop multidrug resistance (6, 7), we found that the majority (63.5%) of the 971 isolates collected from 590 patients were susceptible to all seven universally tested antimicrobials (Fig. 1M and N) and that only 13.8% of isolates were MDR. These frequencies were comparable to those reported in surveillance studies in the United States (24, 40) and the Commonwealth of Virginia (41). Consistent with findings in the literature, patient age, selected patient comorbidities such as CF and diabetes, and prior antipseudomonal antimicrobial prescription were associated with P. aeruginosa clinical isolate resistance to multiple antimicrobials (Fig. 2C) (16, 33–35). Resistance mutations have been found to have various impacts on bacterial fitness and virulence factor production (42–44), and we observed multiple novel associations between virulence-related morphologies and antimicrobial susceptibility (Fig. 2C). Interestingly, a lack of bacterial isolate hemolytic activity was associated with beta-lactam resistance, and the absence of pigment production was associated with amikacin and gentamicin resistance. Additionally, a lack of the mucoid phenotype was associated with tobramycin and ciprofloxacin resistance. While an inverse relationship between a mucoid phenotype and tobramycin resistance has been reported (45), others have found no relationship (46) or increased tobramycin resistance in mucoid isolates (47). Additional evidence suggests that biofilms can still limit tobramycin efficacy even when P. aeruginosa populations within a biofilm are susceptible (48, 49). Together, our findings highlight the complex interconnectivity of patient history, pathogen morphology, and antimicrobial resistance.

While urine/catheter was the second most common source in our collection, P. aeruginosa urinary tract infections (UTIs) are rarely a research focus (9). Urine/catheter isolates were phenotypically similar to skin/wound and non-CF lung/trachea isolates (Fig. 3C). Interestingly, patients with urine/catheter isolates were the most likely of any cohort to have been prescribed only nonantipseudomonal treatments prior to isolate collection (Fig. 4B). This finding reflects the general knowledge that P. aeruginosa is not the predominant cause of UTIs (10, 21, 50). The most common antipseudomonal antimicrobial prescribed prior to urine/catheter P. aeruginosa isolation was ciprofloxacin (Fig. 4E). The frequent use of ciprofloxacin for UTIs (51, 52) potentially explains the increased rate of ciprofloxacin resistance seen in these isolates (Fig. 3D) and motivates the need for more integrated clinical reporting of source-specific antimicrobial prescription histories and susceptibility profiles.

Previous work has established that there is a high level of intrapatient isolate heterogeneity in chronic P. aeruginosa infections (25, 26). We expanded upon this well-appreciated phenomenon by separately quantifying the heterogeneity of susceptibility and morphological profiles. At the individual level, we found that intrapatient isolates with highly conserved susceptibility profiles could display heterogeneous morphological profiles (Fig. 5B) and vice versa (Fig. 5C). At a population level, morphological phenotypes were more variable than susceptibility profiles (Fig. 5E) and the two distance metrics were only weakly correlated (Pearson correlation coefficient = 0.12; *P* < 0.001). Additional research is required to better understand how to prevent and balance these two forms of heterogeneity in chronic P. aeruginosa infections.

There were several limitations of this study that should be noted. First, the rate of patient adherence to antimicrobial therapy in our cohort is unknown. While inpatients likely had reasonably high adherence rates, it is not guaranteed that outpatients took antimicrobials as prescribed, and therefore it is likely that our study overestimates prior antimicrobial use. This limitation additionally prevented us from making more nuanced comparisons across treatment doses, durations, and administration routes. Second, while diabetic status and CF status were the only comorbidities gathered from EHRs, patients without diabetes or CF could have had other comorbid conditions that contributed to their overall health status. It is possible that patients had other risk factors for antimicrobial resistant P. aeruginosa infection that were not recorded in this study. Third, we do not distinguish between cocolonizing and coinfecting organisms, and the true number of organisms coisolating with P. aeruginosa is likely greater than what we report. Fourth, due to the short duration of our study, long-term patient history as well as patient outcomes following P. aeruginosa isolation could not be evaluated. Fifth, sputum sampling captures only a subset of the total population and therefore it should be assumed that P. aeruginosa isolates in the CF lung are more heterogeneous than has been reported. The continued surveillance of isolates from the cystic fibrosis patients in our cohort could help to elucidate the impact of sampling on our observed patterns of intrapatient isolate heterogeneity. Sixth, the relationships identified in our study are correlative rather than causative. Future work should include whole-genome sequencing of our isolate collection to identify underlying mechanisms of action. Finally, the majority of isolates were susceptible to all tested antimicrobials, which consequently limited power in our resistant-isolate studies but may also indicate the an even greater array of phenotypic and risk factor diversity exists among resistant isolates at the larger population scale.

In summary, we have demonstrated the value of surveilling clinical P. aeruginosa isolates both broadly across isolate sources and deeply, considering patient metadata, morphological phenotypes, and antimicrobial susceptibility data. Through our multidimensional approach, we have recapitulated well-established trends in the field and identified novel relationships across a unique combination of data types. The key contributions of this study include (i) the identification of associations between virulence-linked isolate morphologies and antimicrobial resistance, (ii) the observation that P. aeruginosa isolates from the urine/catheter are associated with an increased frequency of ciprofloxacin resistance relative to other non-CF sources, and (iii) the finding that within a patient, isolates with consistent susceptibility profiles can display highly variable morphological profiles and vice versa. Our results motivate the continued clinical consideration of microbiological phenotypes and tracking of source-specific antimicrobial prescription and resistance patterns. We have generated a publicly available high-quality clinical data set and have provided an outline for comparable analyses of other human pathogens which will be of value moving forward.

## MATERIALS AND METHODS

### Study design.

The research objectives of this study were to (i) identify clinical and microbiological factors associated with antimicrobial resistance in a large and variable collection of P. aeruginosa isolates, (ii) identify how antimicrobial susceptibility and morphology of P. aeruginosa isolates vary across bodily sites, and (iii) assess the heterogeneity of P. aeruginosa isolates within and across a large cohort of patients. To address these objectives, we conducted a surveillance study in which we indiscriminately collected P. aeruginosa clinical isolates over a 12-month period. In total, 971 isolates were collected from 590 patients ages 1.5 months to 98 years old who were seen at the UVA hospital from February 2019 to February 2020 (IRB-HSR numbers 21191 and 21949). Samples were collected from both outpatient and inpatient settings.

For each isolate, we recorded antimicrobial susceptibility data, morphological phenotypes (mucoid phenotype, metallic sheen, pigment production, and hemolytic activity), isolate source (lung/trachea, urine/catheter, ENT/sinus, skin/wound, blood, or other), and coisolated organisms. We additionally collected patient-specific metadata, including patient age, sex, specific comorbidities (CF and diabetes mellitus), and all types of antimicrobials prescribed (e.g., oral, inhaled, intravenous, and topical) up to 60 days prior to isolate collection. With the exception of morphological phenotypes, all data types were gathered directly from patient charts in EHRs. There were 29 isolates sampled but excluded from this study and the final collection of 971 isolates because of poor laboratory growth or lack of associated patient medical history or because they were indistinguishable (e.g., identical morphology and susceptibility profiles) from isolates collected on the same day as another isolate from the same patient.

### Acquisition, susceptibility testing, and phenotyping of clinical Pseudomonas aeruginosa isolates.

Susceptibility testing was performed by the Clinical Microbiology Laboratory at the UVA Health System. With limited exception, isolates collected from urine were profiled with Vitek 2 systems (AST-GN70 test cards; bioMérieux, Inc.) and isolates from all other sources were profiled with a Sensititre Aris 2X (Thermo Scientific). Kirby-Bauer disk diffusion was used to assess 26 isolates with mucoid phenotype or growth characteristics, which prohibited other susceptibility methods. In order to compare susceptibility profiles across testing methods, isolates were classified as susceptible, intermediate, or resistant to measured antimicrobials based on established breakpoints for P. aeruginosa from the Clinical and Laboratory Standards Institute (CLSI). All breakpoints were consistent with the 29th edition of CLSI document M100 (53) with the exception of the ciprofloxacin breakpoint, which followed the 28th edition (54, 55). Isolates were considered MDR if they were intermediate or resistant to at least one antimicrobial agent in three or more of the following categories: aminoglycosides, antipseudomonal carbapenems, antipseudomonal cephalosporins, antipseudomonal fluoroquinolones, antipseudomonal penicillins plus beta-lactamase inhibitors, or monobactams (30). Aztreonam susceptibility was considered in assessment of MDR for isolates profiled with a Sensititre Aris 2X (Thermo Scientific).

Morphological phenotyping was performed by a single experimenter throughout the collection period. If a phenotype could not be determined, isolates were regrown and assessed. Phenotypes that were not clearly absent were categorized as present. Clinical isolates on blood agar plates were acquired on a weekly basis from the Clinical Microbiology Laboratory and were immediately visually categorized as hemolytic or nonhemolytic based on their ability to clear blood agar. Presence of a mucoid phenotype or metallic sheen was additionally recorded based on visual colony inspection. Single colonies were selected from blood agar plates, inoculated into 5 ml of lysogeny broth (LB) medium, and incubated overnight at 37°C with shaking (100 to 200 rpm). Liquid cultures were then centrifuged in 15-ml conical tubes (3,000 rpm, 10 min) and the supernatant was aspirated. Pellets of each isolate were resuspended in 50% glycerol mixed with LB medium, aliquoted, and frozen in duplicate at −20°C. Remaining cultures were streaked onto cetrimide agar plates and incubated overnight at 37°C. Growth on cetrimide agar was assessed for pigment production as well as for homogeneity in colony morphology.

### Collection and processing patient metadata.

The following information was collected from patient EHRs for each isolate: age, sex, source, coisolating organisms at the time of sample isolation, whether the patient had CF or diabetes mellitus, and antimicrobial prescription history from the 60 days prior to isolation. For each isolate, age was reported as the patient age on the date of isolate collection (Table S1; Fig. 2). To avoid double counting patients who had birthdays between visits, at the patient level, age was reported as the median age across all isolates collected from an individual patient (Fig. 1B; Table S1). CF and diabetes were selected as specific comorbidities of interest because these conditions have been previously associated with increased susceptibility to infection (56, 57) and can be confidently identified in patient EHRs. A patient was considered to have CF if CF was a listed medical condition in their chart at the time of isolate collection. Diabetic status was determined by searching listed medical conditions, registry membership, hemoglobin A1c levels, prescriptions, and physician notes. Prediabetic patients were classified as nondiabetic. Patients were considered repeat patients from their second sampling date onward. Isolate sources that could not be classified as from the lung/trachea, urine/catheter, ENT/sinus, skin/wound, or blood were grouped as “other.” Coisolating organisms were considered if they were identified on the same day as P. aeruginosa. A full list of coisolating pathogens can be found in Table S4.

10.1128/mSphere.00393-21.8 TABLE S4 Complete list of coisolating pathogens observed in this study. Download Table S4, DOCX file, 0.02 MB. Copyright © 2021 Dunphy et al. 2021 Dunphy et al. https://creativecommons.org/licenses/by/4.0/ This content is distributed under the terms of the Creative Commons Attribution 4.0 International license.

### Processing antimicrobial prescription data.

EHRs were manually screened for antimicrobials prescribed in any form (e.g., oral, inhaled, intravenous, and topical) and for any duration up to 60 days prior to the day each isolate was collected. Antimicrobial prescriptions starting on or after the isolation date are excluded. Prescribed antimicrobials were categorized as antipseudomonal (AP) or nonantipseudomonal (NAP) (Table S3). Antipseudomonal antimicrobials are those that are used individually or in combination with other antimicrobials to treat P. aeruginosa infections. Antimicrobials that P. aeruginosa is intrinsically resistant to were classified as nonantipseudomonal.

Assessing patient compliance for outpatient prescriptions is complicated in that this study was done only through the EHR. For instance, reliance on a fill date and/or a beginning and end date by clinicians provided more confidence in compliance; however, this assessment was dependent on data entry into the EHR. In the event that isolates from the same patient had conflicting antimicrobial prescription histories, discrepancies were resolved as follows. First, if there was a consensus across a majority of isolates, that prescription was selected. Second, if prescription dates were overlapping, consecutive, or if one range was contained within another, the date range was expanded to include the earliest start and latest end dates. Third, open-ended prescriptions from repeat patients were terminated on the date that the prescription was last recorded. Antimicrobial abbreviations are consistent with those generally used by the American Society for Microbiology (ASM) (https://aac.asm.org/content/abbreviations-and-conventions) with the exception of dapsone (DDS), imipenem/cilastatin (IPMC), metronidazole (MTZ), and rifaximin (RIFX), which were excluded from ASM guidelines. Abbreviations, classes, and antipseudomonal activity of all antimicrobials included in this study can be found in Table S3.

### Calculation of adjusted odds ratios and Pearson correlation coefficients.

Adjusted odds ratios were calculated from the coefficients of multiple logistic regression models. Using antimicrobial susceptibility as a response (S = 0, I/R = 1), the following logistic regressions were performed on the complete isolate collection:

$$\begin{array}{l} \text{ln}(\frac{P_{\text{R}\;\text{or}\;\text{I}}}{P_{\text{S}}})\;=\;b\;+\;b_{\text{age}}X_{\text{age}}\;+\;b_{\text{sex}}X_{\text{sex}}\;+\;b_{\text{cf}}{\text{X}}_{\text{cf}}\;+\;b_{\text{db}}X_{\text{db}}\;+\;b_{\text{co-isolating}}X_{\text{co-isolating}}\;\\ +\;b_{\text{APprescription}}X_{\text{APprescription}}\;+\;b_{\text{NAPprescription}}X_{\text{NAPprescription}}\;+\;b_{\text{repeatpatient}}X_{\text{repeatpatient}}\;\\ +\;b_{\text{seasonSummer}}X_{\text{seasonSummer}}\;+\;b_{\text{seasonSpring}}X_{\text{seasonSpring}}\;+\;b_{\text{seasonWinter}}X_{\text{seasonWinter}}\;\\ +\;b_{\text{source}\_\text{lung}\_\text{trachea}}X_{\text{source}\_\text{lung}\_\text{trachea}}\;+\;b_{\text{source}\_\text{urine}\_\text{catheter}}X_{\text{source}\_\text{urine}\_\text{catheter}}\;+\;b_{\text{Hemolysis}}X_{\text{Hemolysis}}\;\\ +\;b_{\text{Mucoid}}X_{\text{Mucoid}}\;+\;b_{\text{Metallic}}X_{\text{Metallic}}\;+\;b_{\text{Pigment}}X_{\text{Pigment}} \end{array}$$

Seven models were built, one to predict susceptibility to each of the following antimicrobials: amikacin, cefepime, ciprofloxacin, gentamicin, meropenem, piperacillin-tazobactam, and tobramycin. All input variables (*X*_input_) were binary (0 = {absent, no, M}, 1 = {present, yes, F}) with the exception of age, which was continuous. Season was separated into three binary variables, and the isolate source was separated into presence or absence in the lung/trachea or urine/catheter. Adjusted odds ratios are reported as exp(*b*_input_) with the exception of age, which was calculated as exp(*b*_age_ × [max(*X*_age_) − min(*X*_age_)]). Logistic regression was performed with the glm function in R.

Pearson correlation coefficients and associated *P* values were calculated with the Hmisc package in R (58). In addition to the variables inputted into logistic regression models, the Pearson correlation coefficients included all isolate sources and seasons. Fractional deviation was defined as (*n*_observed_ − *n*_expected_)/*n*_expected_, where *n*_observed_ was the observed number of isolates resistant to a pair of antimicrobials and *n*_expected_ was the number of isolates that would be expected to be resistant to both antimicrobials if resistance was statistically independent. For each pair of antimicrobials, fractional deviation was used to assess whether there were more (fractional deviation > 0) or fewer (fractional deviation < 0) coresistant isolates in CF and non-CF populations than would be expected assuming statistical independence (Fig. 2B). Coefficients, fractional deviations, adjusted odds ratios, corrected *P* values, and uncorrected *P* values are listed in Data Set S1.

### Distance calculations, clustering, and radar plots.

Gower distances were calculated between isolate pairs with the cluster package in R (59, 60). Gower distance was used due to its ability to handle categorical variables. For distance calculations, all isolate categories were binary with the exception of antimicrobial susceptibility (S, I, or R) and pigment (green, blue, red, mixed, or none) data (Fig. 1N, 3A and B, and 5E; Fig. S4). Distances were calculated on subsets of isolates and variables included in each analysis.

Isolate distances in susceptibility profiles were hierarchically clustered with the complete linkage method (Fig. 1N). PCoA was performed with the ape package in R (61). PCoA is more flexible than PCA in that it can be performed on non-Euclidean distance matrices (36). Negative eigenvalues in PCoA solutions can result from non-Euclidean distance matrices and artificially inflate the percent variance captured by each component. To address this issue, the percent variance was calculated by dividing eigenvalues by the sum of the absolute values of all eigenvalues. Radar plots were generated with the fmsb package in R (62).

### Intrapatient isolate heterogeneity.

Gower distance matrices were calculated for all isolates across phenotypic profiles (mucoid phenotype, metallic sheen, pigment, and hemolysis) and across susceptibility profiles (S/I/R to seven antimicrobials). For patients with multiple isolates, the median of distances between isolates from each patient were calculated. This calculation was repeated for phenotypic distance and susceptibility distance matrices. Median distances were calculated on the upper half of the distance matrices and did not include the diagonals. Two-dimensional densities and histograms of median isolate-isolate distances across patients were plotted (Fig. 5E). The Pearson correlation coefficient was calculated between phenotypic and susceptibility distance matrices for all isolate-isolate pairs across all patients, and the median distances across all isolate-isolate pairs were reported.

### Statistical tests.

All data analyses and statistical tests were performed in R (v4.0.0). Relatedness of isolates across sources was calculated by permutational multivariate analysis of variance (PERMANOVA) with the vegan package in R (63). Nonnormal distributions were compared by Wilcoxon rank sum test (Fig. 4A; Fig. S2). Fisher’s exact test was used to assess the statistical independence of pairwise antimicrobial resistance frequencies (Fig. 2B). Where noted, *P* values were corrected for multiple comparisons with the Benjamini-Hochberg (BH) method (64). *P* values below 0.05 were considered significant.

### Data availability.

All clinical data are available in Data Set S2. Ages over 90 are reported as “>90” to protect patient privacy (Data Set S2). Complete lists of antimicrobials and coisolating organisms identified in this study can be found in Tables S3 and S4. All adjusted odds ratios, Pearson correlation coefficients, fractional deviations, and associated raw and adjusted *P* values are additionally provided (Data Set S1). Deidentified data and code can be found at https://github.com/lauradunphy/clinicalPseudomonas.

10.1128/mSphere.00393-21.10 DATA SET S2 CSV of morphological phenotypes, paired patient metadata, antimicrobial susceptibility data, prescription data, and coisolated organisms for all isolates. Download Data Set S2, CSV file, 1.5 MB. Copyright © 2021 Dunphy et al. 2021 Dunphy et al. https://creativecommons.org/licenses/by/4.0/ This content is distributed under the terms of the Creative Commons Attribution 4.0 International license.
