# Supplementary material for: Multidimensional Clinical Surveillance of Pseudomonas aeruginosa Reveals Complex Relationships between Isolate Source, Morphology, and Antimicrobial Resistance
Source: mSphere. 2021 Jul 14;6(4):e00393-21. doi: 10.1128/mSphere.00393-21 (PMC8386403; doi:10.1128/mSphere.00393-21)
Supplement: TABLE S3 [file msphere.00393-21-st003.docx]

**Table S3. Complete list of antimicrobials considered in this study arranged by antimicrobial class in descending frequency of prescription.**

| **Antimicrobial** | **Abbreviation** | **Category for Logistic Regression** | **# Prescriptions** |
| --- | --- | --- | --- |
| Aminoglycosides | | | |
| Tobramycin | TOB | Antipseudomonal | 205 |
| Amikacin | AMK | Antipseudomonal | 36 |
| Gentamicin | GEN | Antipseudomonal | 20 |
| Neomycin | NEO | Non-Antipseudomonal | 8 |
| Beta-lactams and combination agents | | | |
| Aztreonam | ATM | Antipseudomonal | 157 |
| Cefepime | FEP | Antipseudomonal | 143 |
| Piperacillin/  Tazobactam | TZP | Antipseudomonal | 117 |
| Cefazolin^1^ | CFZ | Non-Antipseudomonal | 98 |
| Ceftazidime^3^ | CAZ | Antipseudomonal | 85 |
| Cephalexin^1^ | LEX | Non-Antipseudomonal | 55 |
| Amoxicillin/  Clavulanic acid | AMC | Non-Antipseudomonal | 48 |
| Meropenem | MEM | Antipseudomonal | 40 |
| Amoxicillin | AMX | Non-Antipseudomonal | 16 |
| Ertapenem | ETP | Non-Antipseudomonal | 13 |
| Ampicillin/  Sulbactam | SAM | Non-Antipseudomonal | 12 |
| Ceftolozane^5^/  Tazobactam | C/T | Antipseudomonal | 12 |
| Cefdinir^3^ | CDR | Non-Antipseudomonal | 9 |
| Cefuroxime^2^ | CXM | Non-Antipseudomonal | 8 |
| Ampicillin | AMP | Non-Antipseudomonal | 7 |
| Nafcillin | NAF | Non-Antipseudomonal | 6 |
| Imipenem/  Cilastatin | IPMC | Antipseudomonal | 4 |
| Penicillin | PEN | Non-Antipseudomonal | 4 |
| Ceftriaxone^3^ | CRO | Non-Antipseudomonal | 3 |
| Cefadroxil^1^ | CFR | Non-Antipseudomonal | 2 |
| Ceftazidime^3^/  Avibactam | CZA | Antipseudomonal | 1 |
| Fluoroquinolones | | | |
| Ciprofloxacin | CIP | Antipseudomonal | 192 |
| Levofloxacin | LVX | Antipseudomonal | 18 |
| Moxifloxacin | MXF | Non-Antipseudomonal | 5 |
| Glycopeptides | | | |
| Vancomycin | VAN | Non-Antipseudomonal | 177 |
| Macrolides | | | |
| Azithromycin | AZM | Non-Antipseudomonal | 261 |
| Clarithromycin | CLR | Non-Antipseudomonal | 6 |
| Other agents | | | |
| Metronidazole | MTZ | Non-Antipseudomonal | 50 |
| Ethambutol | EMB | Non-Antipseudomonal | 22 |
| Tedizolid | TZD | Non-Antipseudomonal | 19 |
| Clindamycin | CLI | Non-Antipseudomonal | 17 |
| Tigecycline | TGC | Non-Antipseudomonal | 14 |
| Rifampin | RIF | Non-Antipseudomonal | 13 |
| Daptomycin | DAP | Non-Antipseudomonal | 9 |
| Trimethoprim | TMP | Non-Antipseudomonal | 5 |
| Dapsone | DDS | Non-Antipseudomonal | 4 |
| Linezolid | LZD | Non-Antipseudomonal | 2 |
| Rifaximin | RIFX | Non-Antipseudomonal | 2 |
| Polymyxins | | | |
| Colistin | CST | Antipseudomonal | 57 |
| Sulfonamides | | | |
| Trimethoprim/  sulfamethoxazole | SXT | Non-Antipseudomonal | 135 |
| Tetracyclines | | | |
| Doxycycline | DOX | Non-Antipseudomonal | 35 |
| Minocycline | MIN | Non-Antipseudomonal | 10 |

^1^ 1^st^ generation cephalosporin

^2^ 2^nd^ generation cephalosporin

^3^ 3^rd^ generation cephalosporin

^5^ 5^th^ generation cephalosporin
