# Supplementary material for: Multidimensional Clinical Surveillance of Pseudomonas aeruginosa Reveals Complex Relationships between Isolate Source, Morphology, and Antimicrobial Resistance
Source: mSphere. 2021 Jul 14;6(4):e00393-21. doi: 10.1128/mSphere.00393-21 (PMC8386403; doi:10.1128/mSphere.00393-21)
Supplement: TABLE S1 [file msphere.00393-21-st001.docx]

**Table S1 – Patient and isolate metadata**

| Variable | # patients (total = 590) | # isolates (total = 971) |
| --- | --- | --- |
| Age, median (IQ25 – IQ75) | 60 (31.1 – 73) | 49 (24 – 68) |
| 0 – 14 years (%) | 65 (11.0%) | 86 (8.9%) |
| 15 – 24 years (%) | 57 (9.66%) | 167 (17.2%) |
| 25 – 44 years (%) | 80 (13.6%) | 202 (20.8%) |
| 45 – 64 years (%) | 143 (24.2%) | 201 (20.7%) |
| 65+ years (%) | 245 (41.5%) | 315 (32.4%) |
| Sex |  |  |
| Female (%) | 305 (51.7%) | 485 (49.9%) |
| Male (%) | 285 (48.3%) | 486 (50.1%) |
| Cystic fibrosis (%) | 106 (18.0%) | 334 (34.4%) |
| Diabetes mellitus* (%) | 158 (26.8%) | 285 (29.4%) |

*Pre-diabetics are not considered to have diabetes
