# Supplementary material for: Multidimensional Clinical Surveillance of Pseudomonas aeruginosa Reveals Complex Relationships between Isolate Source, Morphology, and Antimicrobial Resistance
Source: mSphere. 2021 Jul 14;6(4):e00393-21. doi: 10.1128/mSphere.00393-21 (PMC8386403; doi:10.1128/mSphere.00393-21)
Supplement: TABLE S2 [file msphere.00393-21-st002.docx]

**Table S2 – Breakdown of isolate sources and antimicrobial susceptibility of 971 clinical isolates**

| Variable | # isolates (total = 971) |
| --- | --- |
| Isolate source |  |
| Lung | 443 (45.6%) |
| Urine/Catheter | 242 (24.9%) |
| ENT/Sinus | 144 (14.8%) |
| Skin/Wound | 96 (9.9%) |
| Blood | 26 (2.7%) |
| Other | 20 (2.1%) |
| Antimicrobial susceptibility  (n susceptible) |  |
| Amikacin^†^ | 879 (90.5%) |
| Cefepime ^β^ | 850 (87.5%) |
| Ciprofloxacin^¶^ | 837 (86.2%) |
| Gentamicin^†^ | 842 (86.7%) |
| Meropenem ^β^ | 820 (84.4%) |
| Piperacillin^β^/Tazobactam^‡^ | 792 (81.6%) |
| Tobramycin^†^ | 922 (95.0%) |
| Aztreonam^β^* | 640/751 (85.2%) |

* Not all isolates had susceptibility data available for this antimicrobial

^†^ Aminoglycoside

^β^ Beta-lactam

^¶^ Fluoroquinolone

^‡^ Beta-lactamase inhibitor
