# Supplementary figures and images for: Multidimensional Clinical Surveillance of Pseudomonas aeruginosa Reveals Complex Relationships between Isolate Source, Morphology, and Antimicrobial Resistance
Source: mSphere. 2021 Jul 14;6(4):e00393-21. doi: 10.1128/mSphere.00393-21 (PMC8386403; doi:10.1128/mSphere.00393-21)

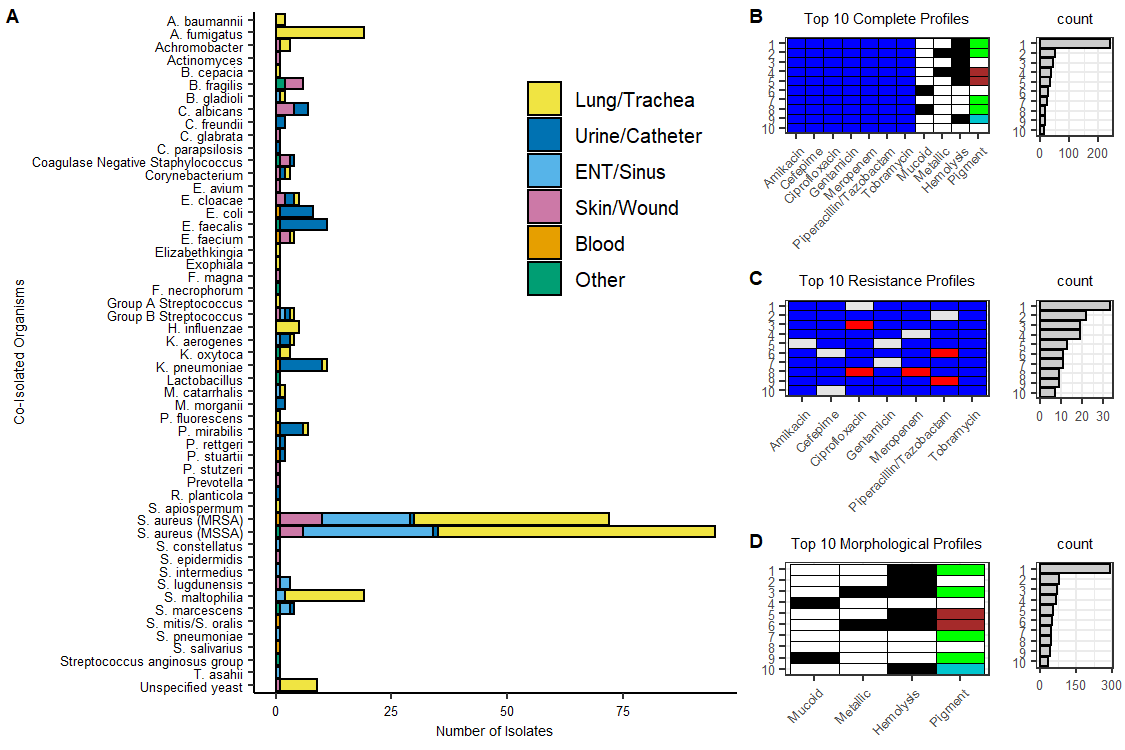

Supplement: FIG S1 [file msphere.00393-21-sf001.tif]

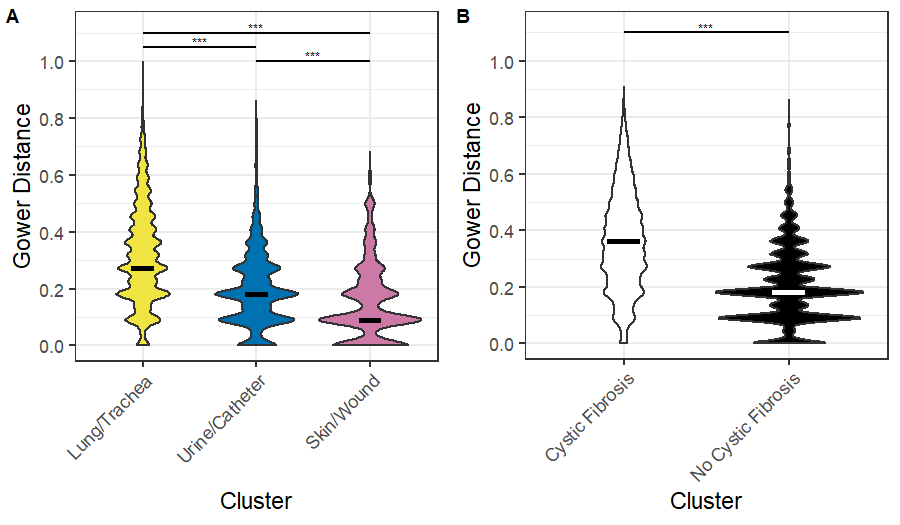

Supplement: FIG S2 [file msphere.00393-21-sf002.tif]

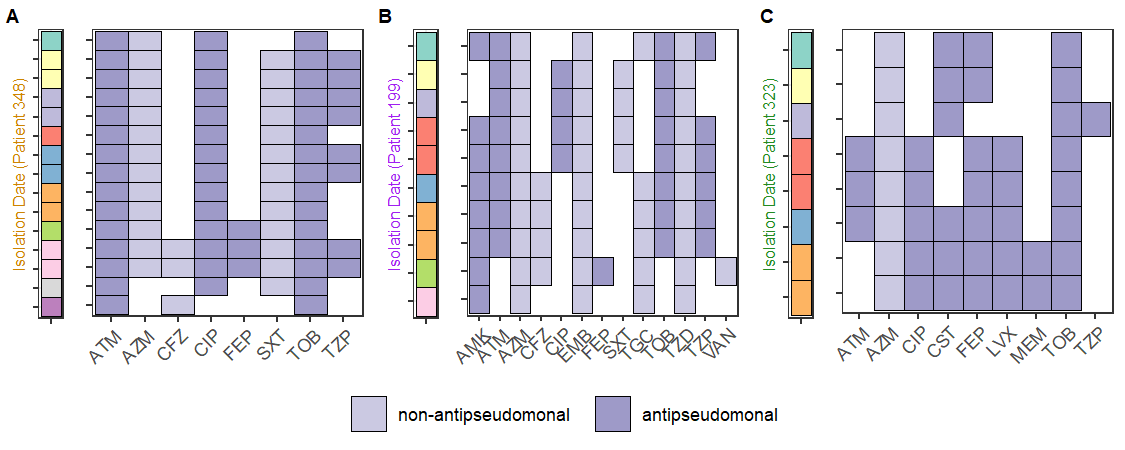

Supplement: FIG S3 [file msphere.00393-21-sf003.tif]

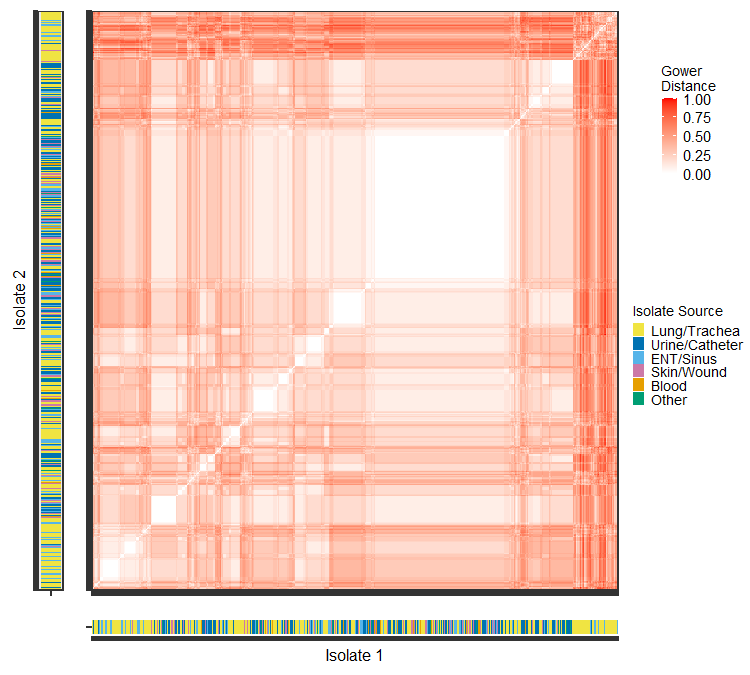

Supplement: FIG S4 [file msphere.00393-21-sf004.tif]
