# Supplementary material for: Multidimensional Clinical Surveillance of Pseudomonas aeruginosa Reveals Complex Relationships between Isolate Source, Morphology, and Antimicrobial Resistance
Source: mSphere. 2021 Jul 14;6(4):e00393-21. doi: 10.1128/mSphere.00393-21 (PMC8386403; doi:10.1128/mSphere.00393-21)
Supplement: TABLE S4 [file msphere.00393-21-st004.docx]

**Table S4. Complete list of co-isolating pathogens observed in this study.**

| Organism | Classification | Number of Occurrences |
| --- | --- | --- |
| Acinetobacter baumannii | Bacteria | 2 |
| *Aspergillis fumigatus* | Fungus | 19 |
| *Achromobacter sp.* | Bacteria | 3 |
| *Actinomyces sp.* | Bacteria | 1 |
| *Burkholderia cepacia* | Bacteria | 1 |
| *B. gladioli* | Bacteria | 2 |
| *Bacteroides fragilis* | Bacteria | 6 |
| *Candida albicans* | Fungus | 7 |
| *C. glabrata* | Fungus | 1 |
| *C. parapsilosis* | Fungus | 1 |
| *Citrobacter freundii* | Bacteria | 2 |
| Coagulase Negative staphylococcus | Bacteria | 4 |
| *Corynebacterium sp.* | Bacteria | 3 |
| *Enterococcus avium* | Bacteria | 1 |
| *E. faecalis* | Bacteria | 11 |
| *E. faecium* | Bacteria | 4 |
| *Enterobacter cloacae* | Bacteria | 5 |
| *Escherichia coli* | Bacteria | 8 |
| *Elizabethkingia* | Bacteria | 1 |
| *Exophiala* | Fungus | 1 |
| *Finegoldia magna* | Bacteria | 1 |
| *Fusobacterium necrophorum* | Bacteria | 1 |
| Group A streptococcus | Bacteria | 1 |
| Group B streptococcus | Bacteria | 4 |
| *Haemophilus influenzae* | Bacteria | 5 |
| *Klebsiella aerogenes* | Bacteria | 4 |
| *K. oxytoca* | Bacteria | 3 |
| *K. pneumoniae* | Bacteria | 11 |
| *Lactobacillus* | Bacteria | 1 |
| *Moraxella catarrhalis* | Bacteria | 2 |
| *Morganella morganii* | Bacteria | 2 |
| *Prevotella* | Bacteria | 1 |
| *Proteus mirabilis* | Bacteria | 7 |
| *Providencia rettgeri* | Bacteria | 2 |
| *P. stuartii* | Bacteria | 2 |
| *Pseudomonas fluorescens* | Bacteria | 1 |
| *P. stutzeri* | Bacteria | 1 |
| *Raoultella planticola* | Bacteria | 1 |
| *Scedosporium apiospermum* | Fungus | 1 |
| Serattia marcescens | Bacteria | 4 |
| *Staphylococcus aureus* (MSSA) | Bacteria | 95 |
| *S. aureus* (MRSA) | Bacteria | 72 |
| *S. epidermidis* | Bacteria | 1 |
| *S. intermedius* | Bacteria | 1 |
| *S. lugdunensis* | Bacteria | 3 |
| *Stenotrophomonas maltophilia* | Bacteria | 19 |
| *Streptococcus anginosus* group | Bacteria | 1 |
| *Streptococcus constellatus* | Bacteria | 1 |
| *S. mitis/S. oralis* | Bacteria | 1 |
| *S. pneumoniae* | Bacteria | 1 |
| *S. salivarius* | Bacteria | 1 |
| *T. asahii* | Fungus | 1 |
| Unspecified yeast | Fungus | 9 |
